# Association among Doctor-Patient Communication, Trust, and Patients' Negative Stereotypes for Healthcare Professionals during COVID-19: A Cross-Sectional Study

**DOI:** 10.1155/2023/5522135

**Published:** 2023-12-26

**Authors:** Yao Wang, Xiaoou Bu, Yanjiao Wang, Yawen Du, Yu Liu, Pei Wang

**Affiliations:** ^1^College of Education, Lanzhou City University, Lanzhou, China; ^2^School of Teacher Education, Honghe University, Mengzi, China; ^3^School of Mental Health, Wenzhou Medical University, Wenzhou, China; ^4^Faculty of Education, East China Normal University, Shanghai, China; ^5^Medical Department, Shanghai General Hospital, Shanghai Jiao Tong University School of Medicine, Shanghai, China

## Abstract

**Aim:**

The purpose of this study was to examine the association among doctor-patient communication, trust, and patients' negative stereotypes for healthcare professionals during COVID-19.

**Background:**

Patients' negative stereotypes for healthcare professionals have been considered a major cause of violence against healthcare professionals. During COVID-19, since the fear for illness could exacerbate patients' negative stereotypes for healthcare professionals, therefore, how to inhibit patients' negative stereotypes for healthcare professionals has become a top priority.

**Methods:**

From January 2020 to April 2020, a cross-sectional survey study was performed in which a total of 2000 patients were convenience-sampled from different regions of China. The survey measured doctor-patient communication, institutional trust, interpersonal trust, and patients' negative stereotypes for healthcare professionals, respectively. After standardizing the total data scores, the association between doctor-patient communication, trust and patients' negative stereotypes for healthcare professionals using the bootstrap method was built.

**Results:**

There was a moderate to the high correlation between doctor-patient communication, patients' negative stereotypes for healthcare professionals, interpersonal trust, and institution trust (*r* = 0.36–0.68, *p* < 0.01). Mediation analysis showed that patients' interpersonal trust (*β* = −0.25, 95% CI: −0.312–−0.209) than patients' institution trust (*β* = −0.02, 95% CI: −0.042–−0.007) played a greater role in suppressing negative stereotypes through doctor-patient communication.

**Conclusions:**

Through communication, healthcare professionals and patients increase their familiarity and identification, thereby reducing patients' negative stereotypes. Moreover, because interpersonal trust connotes emotional and cognitive trust, it was more beneficial than institution trust in reducing negative stereotypes. *Implications for Nursing Management*. The results of this study can tell governments, healthcare organizations, communities, and healthcare professionals that reducing violent behaviors based on negative stereotypes requires attention to institutional trust building, interpersonal trust development, and communication improvement.

## 1. Introduction

In China, the incidence of violence against healthcare workers reached 59.64%–76.2% [1]. In this regard, patients' negative stereotypes for healthcare professionals are identified as the main reason [2]. Stereotypes are defined as fixed, generalized, and abstract perceptions about a certain type of person or thing, and having negative stereotypes can interfere with an individual's cognitive work and lead to negativity and prejudice against the target group [3]. It was found that when patients have negative stereotypes for healthcare professionals, they are less likely to seek care and have fewer patient-provider interactions during the consultation process [4], which ultimately affects patient satisfaction with the providers.

During COVID-19, patients' negative stereotypes for healthcare professionals may be further amplified. Because of the rapid contagiousness and seriousness of COVID-19, patients feel significantly more anxious and out of control [5]; meanwhile, the healthcare sector has implemented a series of policies to reduce the risk of COVID-19 transmission, such as patient isolation, and limiting the number of visits etc., all of which may lead to patients' negative stereotypes for healthcare professionals. Recently, a meta-analysis involving 11,938 healthcare professionals around the world confirmed that there was a general increase in stigma and violence induced by patients' negative stereotypes during COVID-19 [6]. Therefore, there are significant practical implications on how to effectively inhibit patients' negative stereotypes for healthcare professionals.

Intergroup contact theory states that intergroup contact can lead to more positive attitudes and a decrease in negative stereotypes [7]. In which communication, as the main mode of intergroup contact, has an important influence on negative stereotypes. Particularly in China, doctor-patient communication is a key indicator affecting patients' stereotypes for healthcare professionals. China has over 1.4 billion people but less than 5 million doctors [8]. The severely imbalanced doctor-patient ratio has led to a tendency for healthcare professionals to compress communication time with patients, in order to ensure that more patients are served in a given amount of time [9]. In this situation, Chinese healthcare professionals usually focus on disease in the process of communication with patients, neglecting emotional communication and lacking information sharing with patients, thus forming patients' negative stereotypes for healthcare professionals, such as hard attitude and lack of empathy [10, 11]. However, through high-quality doctor-patient communication, it will help convey positive messages and eliminate treatment misunderstandings between doctors and patients, which will improve patients' positive stereotypes for healthcare professionals [12]. Research confirms that by increasing the frequency of doctor-patient communication, patients conveyed more positive messages about healthcare professionals among themselves, while expressing more positive stereotypes for healthcare professionals [13]. In addition, during the communication process, both shared information and joint decision-making also suppressed negative stereotypes for healthcare professionals [14].

Although there is some research support for the effectiveness of doctor-patient communication in reducing patients' negative stereotypes, however, communication as a two-way interactive process, the effect of communication is influenced by the psychosocial aspects of the message sender and the message receiver [15]. For example, in the theory of information transfer, the sender and receiver of information consider the possible costs of sending or receiving information. Therefore, during the transmission of information, the receiver of the message critically evaluates the transmitted information, which in turn affects the effectiveness of the communication [16, 17]. On this account, psychological factors are important drivers for the effectiveness of doctor-patient communication. So, which psychological factors play a key role between communication and negative stereotyping?

It was found that trust is an essential element linking communication and stereotypes [18]. By trust, patients can feel the kindness conveyed in the doctor-patient communication [19], enhancing the patient's identification with the healthcare provider [20]. Besides, patient trust also helps sharing treatment information during communication [21], which in turn influences patients' stereotypes for healthcare professionals. According to Luhmann's (2018) classification of trust, trust is divided into interpersonal trust and institutional trust, which have different roles and psychological mechanisms, respectively. Specifically, interpersonal trust is an expected judgment and psychological state, in which patients and healthcare professionals believe that the other party will not act against them during the interaction [23]. Institutional trust is the expectation and belief whether the healthcare system is credible in the doctor-patient interaction [24]. Compared with interpersonal trust, institutional trust can control people's behavior within a certain range by approving and encouraging behaviors that conform to institutional regulations, and punishing behaviors that violate institutional regulations, hence greatly reducing social uncertainty and risk [25]. Thus, in the medical process, patients are able to demonstrate high interpersonal trust if the healthcare provider is an “acquaintance” or a family member or friend, but if the patient has low interpersonal trust in the healthcare provider, they are likely to seek institutional trust as a substitute [26]. During the effect of doctor-patient communication on negative stereotypes, interpersonal trust and institutional trust also have different psychological mechanisms. On the one hand, interpersonal trust builds mutual emotions and awareness among individuals in interpersonal interactions, which depend on mutual rational thinking and emotional identification [27]. Thus, interpersonal trust contains both cognitive and affective elements [28]. However, institutional trust is built by combining regulations, discipline, and preventive mechanisms, where people's trust in the institution depends mainly on its rationality and legitimacy excluding emotional elements [22]. Therefore, in the effect of doctor-patient communication on stereotypes, interpersonal trust can affect stereotypes through the tone emotion and content cognition in the communication process, but institutional trust only works by influencing the cognitive component of the communication process. Studies confirmed that interpersonal trust relies on influencing listener emotions to enhance the effectiveness of communication during COVID-19 [29], and that institutional trust is dependent on honest and transparent communication content acting on patients' cognitive [30]. On the other hand, interpersonal trust involves interdependence among individuals and promotes cooperation mainly by reducing the fear of individuals being used by others [31]. Once there is a breach of trust in others, individuals tend to pay attention to negative stimuli of others evoking negative stereotypes [32]. By contrast, institutional trust promotes cooperation through deterrence and balance [33]. When people do not trust the system that protects their rights and interests, they become less tolerant of the outgroup, which in turn increases hostility toward the outgroup and creates negative stereotypes of the outgroup [34].

In summary, doctor-patient communication is closely related to patients' negative stereotypes for healthcare professionals, and also, trust as a psychological driver affecting communication and negative stereotypes, with different trust mechanisms playing different roles between communication and negative stereotypes. Especially during COVID-19, the emotional and rational components carried by interpersonal and institutional trust are likely to be further prominent in communication and stereotypes. Based on this, the study investigated doctor-patient communication, interpersonal trust, institutional trust, and patients' stereotypes for healthcare professionals during COVID-19 using a questionnaire method, aiming to further elucidate the association among doctor-patient communication, trust, and patients' negative stereotypes for healthcare professionals.

## 2. Methodology

### 2.1. Sample and Process

From January 2020 to April 2020, a nationwide cross-sectional survey was conducted using a convenience sample at hospitals from 28 provinces in China, including Beijing, Shanghai, Jiangsu, Anhui, Hunan, Xinjiang, and Guangxi, and a total of 2,000 participants were recruited. If the patients were 18 years of age or older, volunteered to participate in the study, and provided verbal informed consent, they were eligible to participate. After completing the research, patients will receive gifts that will be randomly selected. Regarding the survey process, we used a specialized survey platform “Questionnaire Star” for data collection. This platform has stable and reliable system and diversified service functions, which can guarantee the stability of access and data security. At the beginning of the web-based questionnaire, the researchers used standardized instructions about the purpose of the study and informed participants that they could leave the study at any time. Besides, the responses are administered online, allowing patients to fill out the questionnaire at their convenience and have the opportunity to ask the researcher questions. All study data were collected anonymously and confidentially. After the data collection was completed, we performed data cleaning to remove invalid samples, which involved excluding duplicate samples, logically contradictory answers, incorrect values, and clearly inattentive responses. When the data were deemed to have content accuracy, we further excluded data beyond 3 standard deviations of the mean response time, resulting in a total of 1445 valid data. Permission for this study was obtained from the Ethics Committee of Shanghai Normal University.

### 2.2. Questionnaire Measurement

The SEGUE Framework, developed by Makoul [35] and revised by China Medical University in 2006, was used to measure patient ratings for healthcare professionals' communication skills. This scale has five dimensions: preparation, requesting information, providing information, understanding the patient, and ending the consultation. These five dimensions consist of a total of 25 entries, such as “the doctor will greet me politely during the visit” and “the doctor will pick up on my cues.” All questions were scored on a five-point Likert scale (1 = “never” and 5 = “all the time”), with higher scores reflecting better patient ratings for physicians' communication skills. Cronbach's alpha coefficient for the scale was 0.878.

The medical stereotype questionnaire developed by Ye et al. [36] was used to measure patients' stereotypes for healthcare professionals. The scale has a total of 24 items, such as “respect for life” and “professionalism,” all of which are rated on a five-point Likert scale (1 = “completely disagree” and 5 = “completely agree”), with lower scores indicating a stronger patients' negative stereotypes for healthcare professionals. Cronbach's alpha coefficient for this scale was 0.980.

The Chinese version of the Wake Forest Trust Scale (CWFPTS) is used to assess patients' interpersonal trust in their physicians. The scale was originally developed by Hall et al. and was introduced and revised in Chinese in 2012 [37]. The scale consists of 10 items that cover topics such as “my doctor has my best interests at heart” and “I trust my doctor.” All questions were scored on a five-point Likert scale (1 = “strongly disagree” and 5 = “strongly agree”), with higher scores reflecting higher levels of interpersonal trust. Cronbach's alpha for CWFPTS was 0.89.

A self-administered questionnaire was used to measure patients' institutional trust in the healthcare system. The scale assesses patients' trust in the process of access to care, trust in the professional practices of healthcare providers, trust in the quality and safety of the healthcare system, and trust in health insurance. All items were validated by seven front-line medical staff from different regional hospitals using the Delphi method, and there were 31 items, such as “the current medical system is not against social ethics” and “the current medical system reflects fairness.” In addition, all questions were rated on a five-point Likert scale (1 = “completely disagree” and 5 = “completely agree”), with higher scores reflecting greater institutional trust. Cronbach's alpha coefficient for this scale was 0.899.

### 2.3. Analysis

Data analysis was performed using SPSS version 23.0. We performed descriptive analysis, Pearson correlation analysis, and mediation analysis (Model 4 of SPSS Hayes process macro3.3). We normalize all continuous variables and use Hayes' bootstrapping method to test the model. The bootstrapping method is less affected by sample size and does not assume the normality of the mediated paths, so it yields more accurate confidence estimates.

## 3. Results

### 3.1. Sample Characteristics

In this study, 2000 patients were invited to participate voluntarily in the survey. As subjects were allowed to freely choose to withdraw from this study, and also freely choose the time and place of completion, so after we implemented the exclusion criteria in strict accordance with the statistical guidelines of psychometrics and epidemiology, the total number of valid samples recovered was 1445 (72.2%), of which the mean age of the patients was 35.9 years (SD = 11.5). The specific characteristics of the patients are shown in Table 1.

### 3.2. Descriptive Statistics and Correlation Analysis

Table 2 shows all mean scores, standard deviations, and Pearson correlation of the main variables. As expected, doctor-patient communication, interpersonal trust, and institution trust were positively correlated (*r* = 0.36–0.67, *p* < 0.01). The patients' negative stereotypes for healthcare professionals were negatively correlated with doctor-patient communication, interpersonal trust, and institution trust (*r* = −0.36 to −0.68, *p* < 0.01).

### 3.3. Mediation Role Analysis

**Figure 1:**
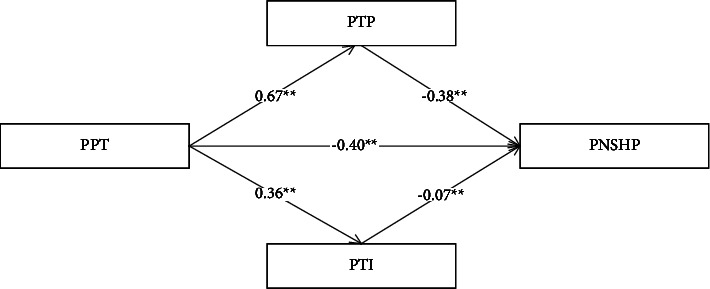
Mediated outcomes of trust in the provider and trust in the institution between communication and negative stereotypes. All paths are expressed as standardized regression coefficients. PPC patients' evaluation of physicians' communication skills; PTP patients' interpersonal trust in their physicians; PTI patients' institutional trust in the healthcare system; PNSHP patients' negative stereotypes of healthcare professionals.

When testing the relationship between doctor-patient communication and patients' negative stereotypes, we found a significant effect of doctor-patient communication on the total effect of negative stereotypes. After adding in interpersonal trust and institution trust, doctor-patient communication had a direct effect on interpersonal trust (*β* = 0.67, *p* < 0.001) and institution trust (*β* = 0.36, *p* < 0.001). Interpersonal trust (*β* = −0.38, *p* < 0.001) and institution trust (*β* = −0.07, *p* < −0.001) had a direct effect on patients' negative stereotypes. Doctor-patient communication (*β* = −0.40, *p* < 0.001) had a direct effect on patients' negative stereotypes (see Figure 1).

Further, the mediating effect was tested using the bias calibration nonparametric percentile Bootstrap method, and the results showed that interpersonal trust and institution trust mediated significantly, with a mediating effect value of −0.28. Specifically, the mediating effect was generated through two mediating chains: first, an indirect effect consisting of communication ⟶ interpersonal trust ⟶ stereotypes (−0.26), with Bootstrap 95% confidence interval not containing 0, indicating a significant mediating effect on interpersonal trust, second, an indirect effect consisting of communication ⟶ institution trust ⟶ stereotypes (−0.02), with Bootstrap 95% confidence interval does not contain 0, indicating a significant mediating effect on institutional trust (see Table 3).

## 4. Discussion

Our main findings suggest that the doctor-patient communication during COVID-19 was the main factor in reducing patients' negative stereotypes for healthcare professionals. Moreover, doctor-patient communication can indirectly influence patients' negative stereotypes through trust, suggesting that patients' trust is an important psychological process that contributes to patients' negative stereotypes. In addition, at the level of patients' trust, interpersonal trust and institution trust played different roles in reducing patients' negative stereotypes. Of these, interpersonal trust has a greater impact on reducing negative stereotypes than institution trust, and it can be argued that developing interpersonal trust between patients and doctors has a greater benefit in reducing patients' negative stereotypes for healthcare professionals.

Specifically, our study found that doctor-patient communication is effective in reducing patients' negative stereotypes. This finding is both consistent with intergroup contact theory and with previously related studies. It was previously found that contact with the target group increased familiarity, mutual knowledge, and information, and increased identification with the outgroup [38], thereby reducing negative stereotypes of the outgroup. Therefore, doctor-patient communication as the main mode of intergroup contact will increase doctor-patient identification by balancing doctor-patient information and thus reduce patients' negative stereotypes for healthcare professionals. Notably, intergroup contact theory suggests that intergroup contact includes more than direct contact interactions, imaginative and online contact can also have a stereotype-reducing effect. For example, Schumann and Moore [39] conducted an 18-month-long intergroup contact intervention, which showed a similar reduction in negative stereotypes when 547 participants interacted with outgroup members by means of synchronous chat or online games. Thus, although the government adopted a policy of social distance maintenance and isolation to prevent the spread of the epidemic during COVID-19, it is still possible to reduce the patients' negative stereotypes for healthcare professionals by using online web technologies, such as mobile devices, mobile apps, and rolling TV news.

We also found that interpersonal trust played a greater role than institution trust in the effect of doctor-patient communication on patients' negative stereotypes for healthcare professionals. In terms of the effect of doctor-patient communication on interpersonal trust and institution trust, doctor-patient communication has a greater contribution to interpersonal trust. This result is supported by previous studies. Previous research suggested that during COVID-19, real-world uncertainty increases and people face both emotional and cognitive stress. In this context, one is more likely to seek advice and trust individuals with whom one feels a sense of similarity or a close relationship [40]. In addition, social identity theory suggests that specific identity perceptions shape an individual's identification with the group, in which he or she lives and that individuals have positive evaluations in in-group members compared to outgroups. When there is no outgroup, shared destiny and similarity among members can also produce in-group identity [41]. As shared destiny is the result of two or more people experiencing congruence in response to external stimuli. Therefore, COVID-19 can be considered a shared destiny between doctors and patients. Accordingly, shared destiny can enhance the intergroup identity between doctors and patients, which in turn increases the interpersonal trust. On the other hand, between trust and patients' negative stereotypes, interpersonal trust has a stronger effect on reducing negative stereotypes. This result can be explained by risk perception theory. Risk perception theory suggests that people's subjective ratings and judgments of risk can trigger their own attitudes and decision-making tendencies [42]. Studies have found that individuals with high-risk perceptions are more likely to exhibit negative attitudes and evaluations of the target group [43], triggering negative stereotypes of the target. Meanwhile, people found that trust can help reduce patients' perception of risk [44]. However, trust from different sources is crucial when patients are assessing the risks of treatment. Hu et al. [45] used a scenario-based experiment to investigate the effect of trust on risk perception to 316 college students, finding that only individuals with high affective trust had high receptivity to risk, while cognitive trust had no effect on risk perception. Indeed, interpersonal trust has both emotional and cognitive dimensions, while institution trust has only cognitive dimensions. Thus, during COVID-19, where risk is uncertain, patients are more likely to reduce negative stereotypes through the affective dimension of interpersonal trust.

Although this study is based on a patient survey during COVID-19, the findings still have a guiding value for the issue of patients' negative stereotypes for healthcare professionals in nonepidemic periods. First, as for the effect of doctor-patient communication on negative stereotypes, patients' negative stereotypes are rooted in the asymmetry of doctor-patient information, which leads to patients' unreasonable expectations and subjective attitudes bias toward healthcare professionals [46]. However, doctor-patient communication is able to balance the information asymmetry between doctors and patients and change patients' unreasonable perceptions about healthcare professionals [47], which leads to the contextual universality in the effect of communication on stereotypes. Second, as for the effect of doctor-patient communication on interpersonal trust and institutional trust, there is still reason to believe that doctor-patient communication has a stronger effect on interpersonal trust than institutional trust during the nonepidemic period, especially in light of the developing concept of “patient-centered communication.” Because “patient-centered communication” emphasizes respect, empathy, and values between patients and doctors, which include positive emotions and attitudes [48], therefore, “patient-centered communication” is more conducive to the development of interpersonal trust between doctors and patients. Finally, as for the suppressive effect of interpersonal and institutional trust on negative stereotypes, interpersonal trust may still have played a greater role than institutional trust during the nonepidemic period. Because the healthcare situation is a risky environment with uncertainty, compared to institutional trust, interpersonal trust can further reduce patients' psychological defenses and enhance the shared value between doctors and patients through emotional connection [49]. Meanwhile, interpersonal trust has positive emotions which contribute to a more positive evaluation about others [50]. Accordingly, interpersonal trust remains more influential than institutional trust for negative stereotypes in nonepidemic periods.

There are several shortcomings in our study. First, this study used an online platform to distribute the questionnaire during COVID-19, so the survey sample mainly came from urban patients with developed online information and better medical conditions, which makes the results not generalized to all patient groups (e.g., patients in remote mountainous areas); however, considering the limitations of people mobility during the period of COVID-19, it will be a challenge to extend the coverage for more patient groups in the future during the period of major epidemics, which may require multisectoral cooperation among the government, healthcare sector, and scientific research institutes, and a combined online and offline method was used for questionnaire surveys. Second, the age of the patients in this study is concentrated between 18 and 40 years old, and less patients are older than 60 years old, which may be due to the fact that older patients cannot make good use of mobile devices, so the development of an intelligent web survey system faces challenges in the future, which requires a web survey system that can optimize the presentation of the questionnaire according to the reading habits and operating modes of different patients in the mobile smart terminals, offering a “user-friendly feedback interface,” and providing intelligent auxiliary explanations for the corresponding groups. Finally, this study used a cross-sectional study, which has limitations on causal inferences; therefore, there are challenges in the future to provide more reliable causal evidence on communication, trust, and negative stereotypes, which may require the involvement of more rigorous experimental methods or longitudinal research methods.

In conjunction with the results of this study, we propose the following recommendations for inhibiting patients' negative stereotypes for healthcare professionals: first, doctor-patient communication is an important factor in reducing patients' negative stereotypes; therefore, it is necessary to train medical providers in communication skills by holding training sessions and standardizing the interpretation of questions. Besides, it is important to emphasize the appropriate emotional expression of medical staff and actively promote the sharing of information between doctors and patients [51]. Second, interpersonal trust and institutional trust both contribute to the reduction of negative stereotypes. In response, on the one hand, efforts should be made to develop interpersonal trust. For example, developing physicians' trustworthy traits such as competence, integrity, kindness, etc., and guiding patients to make proper attributions for negative events. On the other hand, attention should be paid to the building of institutional trust. In this regard, governmental departments should supervise the impartiality of the medicine institution design and facilitate the effective implementation of the medical institution, while medical organizations should ensure the openness and transparency of the institution to satisfy patients' security and to reduce their fear of epidemics, etc. Besides, it is important for communities to actively popularize the advocacy of the healthcare institution for further enhancing patients' understanding and recognition of the healthcare institution.

## 5. Conclusions

This study found that doctor-patient communication helped reduce patients' negative stereotypes for healthcare professionals; meanwhile, trust was an important psychological mechanism for communication to influence negative stereotypes, in which interpersonal trust played a greater role than institutional trust. These results also contributed to the reduction of negative stereotypes for healthcare professionals during the nonepidemic period. The government and the medical sector can provide an environment guarantee for institutional trust by improving institutional construction. The community and media can construct a culture environment for interpersonal trust by promoting and strengthening the image of healthcare professionals. Healthcare professionals can further enhance patients' interpersonal trust by developing “patient-centered communication.”

## Figures and Tables

**Table 1:** Demographic characteristics of the patients.

Demographic variables		*n*	(%)
Gender	Male	659	45.6
	Female	786	54.4

Age	18–30	606	41.9
	31–40	416	28.8
	41–50	216	14.9
	51–60	169	11.7
	>60	38	2.6

Education	Primary school or below	55	3.8
	Junior/Senior high school	399	27.6
	College	902	62.4
	Postgraduate	89	6.2

Hospital grade	Tertiary hospital	1037	71.8
	Secondary hospital	161	11.1
	Primary care institution	34	2.4
	Have no idea	213	14.7
	Total	1445	100.0

**Table 2:** Mean, standard deviations, and correlations of the main variables.

	*M* ± SD	1	2	3	4
(1) PPC	94.52 ± 16.13	1			
(2) PTP	30.12 ± 4.16	0.67^*∗∗*^	1		
(3) PTS	80.83 ± 9.80	0.36^*∗∗*^	0.48^*∗∗*^	1	
(4) PNSHP	49.97 ± 12.94	−0.68^*∗∗*^	−0.68^*∗∗*^	−0.39^*∗∗*^	1

*N* = 1445.PPC patients' evaluation of healthcare professionals' communication skills; PTP patients' interpersonal trust in their healthcare professionals; PTI patients' institutional trust in the healthcare system; PNSHP Patients' negative stereotypes for healthcare professionals. ^*∗∗*^*p* < 0.01.

**Table 3:** Mediating effects of interpersonal trust and institution trust between communication and negative stereotypes.

	Effect	BootSE	BootLLCI	BootULCI	Proportion (%)
Total indirect effect	−0.28	0.02	−0.33	−0.23	41.20
Indirect effect1	−0.25	0.02	−0.31	−0.20	37.63
Indirect effect2	−0.02	0.01	−0.04	−0.00	3.55

BootSE, BootLLCI, and BootULCI, respectively, refer to the standard error of indirect effect estimated by the percentile Bootstrap method with deviation correction, the lower limit, and the upper limit of 95% confidence interval.

## Data Availability

The data that support the findings of this study are available from the corresponding author upon reasonable request.
